# Antiretroviral Treatment in Resource-Limited Settings 2012

**DOI:** 10.1155/2012/346708

**Published:** 2012-07-25

**Authors:** Ann Duerr, Fredrick L. Altice, Anthony D. Harries, Annette H. Sohn

**Affiliations:** ^1^HIV Vaccine Trials Network, Fred Hutchinson Cancer Research Center, Seattle, WA 98109, USA; ^2^Vaccine and Infectious Disease & Public Health Science Divisions, Fred Hutchinson Cancer Research Center, Seattle, WA 98109, USA; ^3^Departments of Global Health and Epidemiology, University of Washington, Seattle, WA 98109, USA; ^4^Infectious Diseases Section, AIDS Program, Yale University School of Medicine, New Haven, CT 06510-2283, USA; ^5^Division of Epidemiology of Microbial Diseases, Yale School of Public Health, New Haven, CT 06520-8034, USA; ^6^International Union Against Tuberculosis and Lung Disease (The Union), Winchester SO 21 1TQ, UK; ^7^TREAT Asia/amfAR-The Foundation for AIDS Research, Bangkok 10110, Thailand

Just over 30 years after the beginning of the HIV epidemic, there is evidence that the global response is starting to catch up with the epidemic, at least in some places. This scaling up and treatment response has made enormous progress in providing access to care for HIV-infected persons, even in the least developed countries (LDCs). This special issue examines the current state of HIV care in resource-poor settings. The papers included in this issue examine questions central to expanded treatment in resource-poor settings such as access to antiretroviral therapy (ART), adherence and retention, management of HIV-associated cancers and opportunistic infections (OIs), and treatment complications. The issue also addresses arenas where progress has been slower than anticipated, such as the prevention of mother-to-child HIV transmission (PMTCT) and the need for more effective tuberculosis (TB) integration and TB treatment campaigns for people living with HIV/AIDS (PLWHA).

The global effort to make ART available to all HIV-infected persons has made great progress in the past decade. By the end of 2010, 6.65 million people were receiving ART globally [1]. Impressive as this number appears, it woefully represents less than half of those who need treatment. The scale of the epidemic and the response to it demand expanded and innovative responses from international organizations, national governments, care providers, and PLWHA themselves. Among the questions that must be answered as the HIV treatment world transitions from management of a crisis to management of a chronic disease are how will care be maintained and expanded in an era of decreasing donor funding? [2, 3] How can programs be most appropriately tailored for local epidemics, especially those that are expanding rapidly? How can the HIV care continuum (HIV testing, linkage to care, adherence, and retention) be strengthened and integrated into other local health services provision?

Four papers in this issue address these broad themes. One examines the potential role of PLWHA as “expert patients” who provide psychosocial and adherence support as well as provision of ART to stable patients (*“Are expert patients an untapped resource for ART provision in Sub-Saharan Africa*?” by T. Decroo et al.). Results from a study in Kenya and programmatic data from Mozambique suggest that this approach can support high retention in care while dramatically reducing clinic burden. Two other studies examine the HIV care continuum in 18 sites in the Asia-Pacific region (“*Loss to followup in HIV-infected patients from Asia-Pacific region: results from TAHOD*” by J. Zhou et al.) and 10 treatment programs in Central Africa (*“Older adults accessing HIV care and treatment and adherence in the IeDEA Central Africa cohort”* by J. Newman et al.). Regional and local analyses of such data will be important to identify individuals, such as younger patients, at risk for poor adherence and loss to follow-up and ultimately provide effective solutions. A fourth study (*“Short-term rationing of combination antiretroviral therapy: impact on morbidity, mortality, and loss to follow-up in a large HIV treatment program in western Kenya”* by A. J. Bell et al.) retrospectively demonstrates increased mortality and loss to follow-up during a 6-month interruption in ART supply—a cautionary note to program officials and practitioners about the crucial need for drug forecasting, timely drug procurement, and efficient drug distribution as key priorities. Mitigation of the potential threat that reduced funding poses to the benefits gained to date will require multilateral efforts, integration of prevention and treatment efforts, reallocation of health-related human resources, and access to less costly medications and interventions. 

Although HIV-related mortality was reduced for the first time globally in 2009 [4], a number of medical conditions continue to fuel HIV-related morbidity and mortality among PLWHA. For those not accessing ART, OIs and malignancies continue to dominate complications from HIV. For those who access ART, increased age-matched morbidity and mortality persist related to a number of conditions, including TB, end-stage liver disease, renal and neurological deterioration, non-AIDS-associated malignancies, and lipodystrophic syndromes. In this issue, H. Francis et al. describe how treatment of endemic Kaposi's sarcoma can be provided in LDCs in Sub-Saharan Africa, yet mortality remains high and underscores the need for palliative care (*“A prospective study assessing tumour response, survival, and palliative care outcomes in patients with HIV-related Kaposi's sarcoma at Queen Elizabeth Central Hospital, Blantyre, Malawi” by *H. Francis et al.). Achhra et al. demonstrate that ART regimens differ between LDCs and resource-rich regions. Such regimens utilized in LDCs may ultimately impact long-term complications of ART, including hyperlipidemia, which in turn may influence the development of cardiovascular-related morbidity and mortality (*“Differences in lipid measurements by antiretroviral regimen exposure in cohorts from Asia and Australia”* by A. C. Achhra et al.).

At a global level, HIV-related mortality has been reduced by 19% overall [4], but these effects have not been experienced uniformly. For example, HIV-related mortality increased by 24% in Eastern Europe and Central Asia where the epidemic is largely concentrated among people who inject drugs, which are primarily opioids [5]. Unlike the laudatory successes of ART rollout in the most affected regions of Sub-Saharan Africa, ART coverage in these regions remains less than 5% [6]. In such settings, medically treatable comorbid conditions, including alcohol use disorders and opioid dependence [5], remain untreated and are critical to optimize early HIV diagnosis, entry into care, ART initiation, and ART adherence [5, 7]. In this issue, M. G. Neuman et al. systematically review the negative impact of alcohol use disorders on ART adherence and other adverse consequences (*“Alcohol consumption, progression of disease and other comorbidities, and responses to antiretroviral medication in people living with HIV”* by M. G. Neuman et al.). Indeed, mathematical models suggest that the most effective and cost-effective method to reduce new HIV infections among injection drug users is to provide effective treatment for the underlying substance use disorder [8]. 

Not only do ART access disparities persist geographically but also remain problematic for pregnant women and children. Cumulative and incident cases of global pediatric HIV infections reflect ongoing difficulties in implementing a range of effective perinatal prevention interventions. The individual failures at each step of the “PMTCT cascade” of care from pregnancy to early infancy resulted in only 28% of HIV-exposed infants receiving an HIV test by the age of two months [9]. The three pediatric studies in this issue help to show the consequences of these gaps in care and the potential for closing them. 

Even in major referral “centers of excellence” in Uganda, children continue to present at older ages with advanced clinical and immunologic disease (*“Barriers to initiation of pediatric HIV treatment in Uganda: a mixed*-*method study”* by T. S. Boender et al.). In order to identify them before they develop complications of HIV such as malnutrition (*“Challenges in the management of HIV-infected malnourished children in Sub-Saharan Africa”* by I. Trehan et al.), we need broader implementation of more strategic approaches, such as that reported in Kenya (*“Towards elimination of mother-to-child transmission of HIV: the impact of a rapid results initiative in Nyanza Province, Kenya”* by L. L. Dillabaugh et al.). The Kenya example demonstrates how a local assessment and tailored intervention led to significant improvements in uptake of maternal and infant ART prophylaxis. Given that ART coverage among the estimated 2 million children who need it is only 23%—less than half that of adults—finding better solutions to both prevent new infections and refer infants immediately into testing and care is imperative. 

Another arena that needs more focused attention is the interface between TB and HIV. In 2010, there were an estimated 1.1 million adults and children with HIV-associated TB of whom 350,000 died [10]. Just over 80% of this morbidity and mortality occurs in Sub-Saharan Africa, with 9 countries in the southern part of the continent accounting for over half of the burden of disease [10]. Recognized strategies to reduce the dual burden of disease include the following: (1) preventing TB in HIV-infected persons through the “Three I's” (intensified case finding, infection control, and isoniazid preventive therapy) and early ART initiation and (2) providing HIV care and treatment for coinfected TB patients through HIV testing, with cotrimoxazole preventive therapy and timely ART for those diagnosed with HIV. 

This supplement features two additional papers from Kenya that partly address these issues. R. Granich et al. (*“Achieving universal access for human immunodeficiency virus and tuberculosis: potential prevention impact of an integrated multi-disease prevention campaign in Kenya” *by R. Granich et al.”) report on a community-based multidisease prevention campaign targeting 5000 people with an intervention incorporating HIV counseling and testing, condoms, insecticide-treated bed nets, and water filters. There was a high uptake of HIV testing, and of those testing HIV positive, the CD4-lymphocyte counts were significantly higher than in patients admitted and HIV-tested at a district hospital. The authors project the potential HIV and TB prevention impact of this campaign strategy for different ART initiation scenarios, concluding that such campaigns could significantly contribute to TB prevention efforts in high-burden countries. D. N. Shaffer et al. (*“Successes and challenges in an integrated tuberculosis/HIV clinic in a rural, resource-limited setting: experiences from Kericho, Kenya”* by D. N. Shaffer et al.”) show that even in a rural, resource-limited district hospital it is possible to run an integrated HIV/TB clinic and implement recommended interventions, all of which led to improved treatment outcomes and reduced mortality during a five-year period.

In summary, enormous progress has been made in the last 8 years with the scale-up of ART in low- and middle-income countries. WHO data document that over 6.5 million people accessed this treatment by the end of 2010; without this intervention, often provided primarily to those with advanced immunodeficiency, the lives of millions of PLWHA would have been lost. There is much, however, left to do. ART expansion is urgently needed in several regions globally, and where there are current success stories, ART needs to be sustained. This is crucially dependent on secure, regular, and timely financial resources and greater national commitment to domestic health programs. The authors who contributed to this issue and their counterparts in the global HIV/AIDS treatment effort remind us that continued commitment to successful strategies and dedicated searches for solutions to the ongoing challenges and obstacles will be necessary if we are to achieve an “AIDS-free generation.”

*Ann Duerr* *Frederick L. Altice* *Anthony D. Harries* *Annette H. Sohn*
